# Genome-Wide SNP Analysis of Southern African Populations Provides New Insights into the Dispersal of Bantu-Speaking Groups

**DOI:** 10.1093/gbe/evv164

**Published:** 2015-09-11

**Authors:** Miguel González-Santos, Francesco Montinaro, Ockie Oosthuizen, Erica Oosthuizen, George B.J. Busby, Paolo Anagnostou, Giovanni Destro-Bisol, Vincenzo Pascali, Cristian Capelli

**Affiliations:** ^1^Department of Zoology, University of Oxford, United Kingdom; ^2^Institute of Legal Medicine, Catholic University, Rome, Italy; ^3^School of Medicine, University of Namibia, Windhoek, Namibia; ^4^Wellcome Trust Centre for Human Genetics, Oxford, United Kingdom; ^5^Dipartimento di Biologia Ambientale, Università “La Sapienza”, Rome, Italy; ^6^Istituto Italiano di Antropologia, Rome, Italy

**Keywords:** Southern Africa, Bantu speakers, admixture, genome-wide SNPs

## Abstract

The expansion of Bantu-speaking agropastoralist populations had a great impact on the genetic, linguistic, and cultural variation of sub-Saharan Africa. It is generally accepted that Bantu languages originated in an area around the present border between Cameroon and Nigeria approximately 5,000 years ago, from where they spread South and East becoming the largest African linguistic branch. The demic consequences of this event are reflected in the relatively high genetic homogeneity observed across most of sub-Saharan Africa populations. In this work, we explored genome-wide single nucleotide polymorphism data from 28 populations to characterize the genetic components present in sub-Saharan African populations. Combining novel data from four Southern African populations with previously published results, we reject the hypothesis that the “non-Bantu” genetic component reported in South-Eastern Africa (Mozambique) reflects extensive gene flow between incoming agriculturalist and resident hunter-gatherer communities. We alternatively suggest that this novel component is the result of demographic dynamics associated with the Bantu dispersal.

## Introduction

The genetic structure of African populations is the result of both ancient and more recent episodes of migration and admixture (Tishkoff et al. 2009; Hellenthal et al. 2014; Pickrell et al. 2012, 2014). Among these historical events, the expansion of Bantu-speaking agropastoralist populations had a substantial impact on the continental distribution of genetic diversity. Bantu languages (part of the Niger-Congo linguistic group, NC) are thought to have originated near the present border between Cameroon and Nigeria approximately 5,000 years before present (Newman 1995; Diamond and Bellwood 2003). From its place of origin, this branch of the NC linguistic phylum spread East and South across sub-Saharan Africa, together with agricultural techniques and the use of iron (Newman 1995; Diamond and Bellwood 2003). Today, Bantu is the largest African linguistic family in both geographical extension and number of speakers, indicative of the impact this migration had on the continent (de Filippo et al. 2012). However, although there is a general consensus on the place and time of the origin of this movement, the actual routes used are still under debate, with two main hypotheses having recently been tested. These hypotheses differ mainly on the geographical and chronological dimensions of the split between East and West Bantu-speaking groups (de Filippo et al. 2012; Currie et al. 2013). Together with the route followed, another important aspect to be considered is the degree of interaction that populations involved in this expansion had with the groups inhabiting the regions they were moving into. For example, the arrival of Bantu-speaking people in the southern regions of the continent could have led either to isolation or to admixture with the inhabitant pastoralists and hunter-gatherers (Destro-Bisol et al. 2004; Mitchell et al. 2008; Mitchell 2009; de Filippo et al. 2012; Patin et al. 2014).

**Fig. 1.—:**
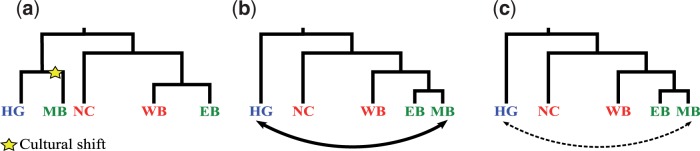
Alternative scenarios for the Mozambique Bantu (MB) differentiation: (*a*) Cultural shift after a split from a non-Bantu, hunter-gatherer population (HG), (*b*) substantial gene flow into a Bantu-speaking group, (*c*) differentiation of an “Eastern” Bantu component (EB, including the MB population), coupled or not with limited gene flow. NC, Niger-Congo; WB, Western-Bantu.

Previous investigations have focused on the demographic dynamics of the Bantu dispersal (Barbieri, Vicente, et al. 2013; Schlebusch et al. 2013; Barbieri et al. 2014; Patin et al. 2014; Pickrell et al. 2012, 2014). Within this context, the differentiation of a population sample from Mozambique from other African populations suggested the presence of a specific South-Eastern component within the continent. This component, found at a proportion higher than 50%, has been interpreted as derived from an ancestral population (presumably related to hunter-gatherer populations) inhabiting the area before the arrival of expanding Bantu speakers (Sikora et al. 2011). The predominance of such a component in extant populations could be explained by the cultural shift of a foraging community (fig. 1*a*) or substantial gene flow into the arriving Bantu-speaking groups (fig. 1*b*). Alternatively, the reported Mozambique uniqueness could simply be the result of the dispersal process whose genetic signature would be expected to be shared with populations related to those living in Mozambique. This scenario might include or not some degree of gene flow from hunter-gatherer populations (fig. 1*c*).

**Fig. 2.—:**
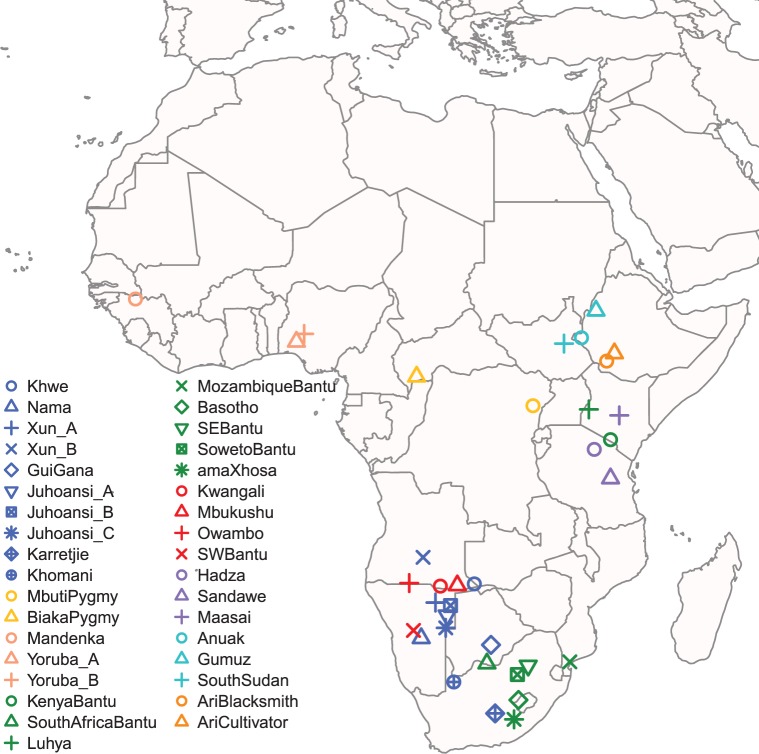
Map of the populations analyzed. The colors indicate different population groups: Blue, Khoisan speakers; yellow, Pygmies; salmon, non-Bantu Niger-Congo speakers; green, East-Bantu speakers; red, West-Bantu speakers; purple, language isolates; turquoise, Nilo-Saharan; and orange, Afro-Asiatic. Information on the populations included can be found in the supplementary material, Supplementary Material online.

To test these hypotheses and further characterize the reported Mozambican component, we analyzed new genome-wide single nucleotide polymorphism (SNP) array data from 35 sub-Saharan African populations from 868 individuals (including 33 novel genotypes from Namibia and Lesotho) (fig. 2). Our results place the so-called “South-Eastern component” within the broader genetic variation of Southern Africa. Additionally, we find no evidence in the Mozambican population for substantial gene flow between Bantu and hunter-gatherer populations from this region.

## Results

**Fig. 3.—:**
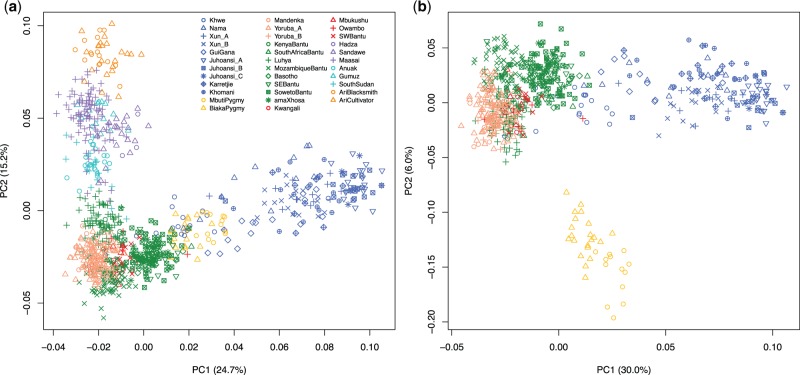
Principal Components (PC) plots for the first and second components: (*a*) Full data set; (*b*) Data set excluding Afro-Asiatic, Nilo-Saharan, and language isolates. Symbols and colors as in figure 2: Blue, Khoisan speakers; yellow, Pygmies; salmon, non-Bantu Niger-Congo speakers; green, East-Bantu speakers; red, West-Bantu speakers; purple, language isolates; turquoise, Nilo-Saharan; and orange, Afro-Asiatic.

**Fig. 4.—:**
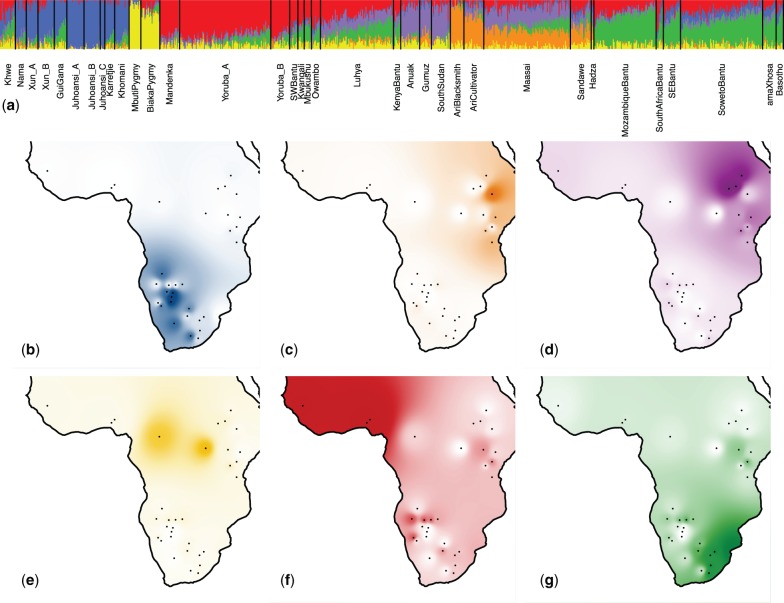
(*a*) ADMIXTURE plot for *K* = 6; (*b–g*) interpolation maps of the ADMIXTURE components for *K* = 6. Colors of the components on the maps are the same as in the ADMIXTURE plot.

Relationships among individuals were explored using principal component analysis (PCA) (fig. 3). The first component (PC1) shows a separation of Khoisan-speaking groups from the other populations, with East non-NC populations separating along the second component (PC2) (fig. 3*a*). To further analyze these results, a second PCA was performed removing the East non-NC (Afro-Asiatic, Nilo-Saharan, and linguistic isolates) populations. In this, the Khoisan-speaking groups and the NC-speaking populations are separated along the first component (PC1), the rainforest hunter-gatherers (RHG, also known as Pygmies) located among the two (fig. 3*b*). RHG are separated from the other populations along the second component (PC2). The NC speakers are spread along the same axis, the Mandenka and Yoruba individuals on one side and various South-Eastern Bantu speakers on the other (fig. 3*b*). We then performed ADMIXTURE analysis, and obtained for *K* = 6 the most suitable number of ancestral components (supplementary fig. S1, Supplementary Material online). Five groups emerge that we associate for simplicity to the linguistic group spoken by the majority of the populations characterized by the indicated component: Khoisan (here considered as Southern African populations speaking click-rich languages; Güldemann and Fehn 2014) (blue), Nilo-Saharan (East-1; purple) and Afro-Asiatic (East-2; orange) in East Africa, RHG (yellow), and NC speakers, the latter being characterized by two major components (red and green in fig. 4*a*). These two components are present in all NC populations but their amount varies among groups. One of these components (red) dominates populations speaking West Bantu and other NC languages, whereas the other one (green) is more common among South-Eastern Bantu speakers (fig. 4*a*). The main component of the Mozambican sample (green) is similarly present in populations from Lesotho and South Africa (even for higher values of *K*, supplementary fig. S5, Supplementary Material online). Notably, the Basotho from Lesotho and the amaXhosa/SE Bantu/SowetoBantu from South Africa also show a non-Bantu component (Khoisan) but additional components (Khoisan or others) are virtually absent in samples from Mozambique. The spatial distribution of these components was visualized by interpolation maps (fig. 4*b–g*). The two main components present in NC populations mapped on to different geographic regions, so that these two can be broadly identified as NC-West and NC-Southeast (fig. 4*f* and *g*, respectively). The Afro-Asiatic and Nilo-Saharan components are mostly restricted to populations from Central-East Africa (fig. 4*c* and *d*) and the RHG component was geographically restricted to Pygmy populations (fig. 4*e*).

The genetic distances among the six components were estimated through *F*_ST_. The largest values are observed when the Khoisan component is compared with the East Africa ones, followed by the comparisons of the first with both components from NC populations. The two NC components (West and East) are the most genetically close, their *F*_ST_ value (0.042) at least two times smaller than any other comparison (table 1).

**Table 1:** *F*_ST_ between ADMIXTURE Components at *K* = 6

	NC-East	NC-West	East-1	East-2	Pygmies	Khoisan
NC-East	—					
NC-West	0.042	—				
East-1	0.099	0.081	—			
East-2	0.123	0.109	0.110	—		
Pygmies	0.097	0.095	0.132	0.137	—	
Khoisan	0.129	0.132	0.165	0.158	0.102	—

Although STRUCTURE-like analyses are useful to identify patterns of gene flow between groups, they are not a formal test of admixture as similar profiles can be the result of several different population histories. For this reason, we carried out *f3* tests (Reich et al. 2009) for windows of 100 SNPs between all the possible trios of populations in our data set and reported all the comparisons in supplementary figure S6 and tables S2 and S3, Supplementary Material online. As expected, this analysis identified most of the known admixture events that characterized African populations, involving Khoisan and Bantu populations in Southern Africa. Interestingly, despite the low number of markers used, the analysis identified East African ancestry in six (out of seven) Khoisan populations, as previously reported (Pickrell et al. 2012). On the other side, the analysis did not find signature of admixture in Karretjie, Owambo, and Kwangali since significant values of the statistics were not observed. However, even though not significant, for these three populations we observed negative values of *f3* statistics 20, 16, and 38 times, respectively. No signature of admixture was found in Mozambique since no statistically significant values of *f3* statistics were observed. In addition, contrary to what reported for Karretjie, Owambo, and Kwangali, none of the *f3* involving Mozambique as target has nonsignificant negative values.

Given the low number of markers analyzed, we performed a cross-validation (CV) *f3* analysis in 1,900 resampled samples composed by different ancestry of Yoruba and of Juhoansi, Sandawe, and Mbuti, as described in the Materials and Methods section. The *f3* statistics for all the simulated samples were always significant, with the exception of Yoruba95%Juhoansi5% and Yoruba95%Mbuti5% where the tests characterized by a *Z*-score lower than −3 were, respectively, 99% and 98%. However, it must be stressed that even for the nonsignificant tests the *Z*-scores were always below −2 (Juhoansi: −2.19; Mbuti: −2.98, −2.90).

**Fig. 5.—:**
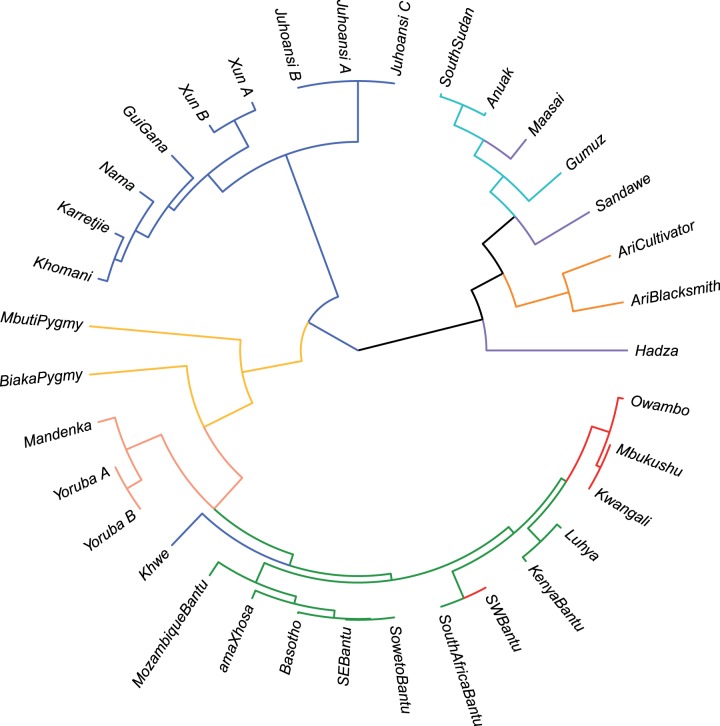
Hierarchical population tree based on pairwise *F*_ST_ values. Color of the branches corresponds to the color of the populations in figure 2.

The pairwise *F*_ST_ population tree shows a separation of the main groups present in our data set, first with a group of Afro-Asiatic, Nilo-Saharan and linguistic isolates, then the Khoisan speakers (except the Khwe, a highly admixed population as can be seen in fig. 4*a*) and the NC-speaking populations (fig. 5), with the RHG located between the last two. In line with what is observed for the ADMIXTURE components, the South-Eastern populations grouped with the other NC populations, the internal branching order mirroring the West–East–Southeast linguistic subdivisions (Currie et al. 2013).

## Discussion

The spread of Bantu-speaking groups across Southern Africa has significantly shaped the distribution of genetic variation of Sub-Sahara Africa (Tishkoff et al. 2009). However, such dispersal did not occur in a void, as hunter-gatherers and pastoralists were already present in the areas that Bantu-speaking farmers were entering. The interactions between residents and newcomers were different in different areas, resulting in different degrees of integration and admixture between these groups (Mitchell 2002; Barbieri, Butthof, et al. 2013; Barbieri et al. 2014; Marks et al. 2014; Patin et al. 2014; Pickrell et al. 2012, 2014). Archaeological and genetic data suggest that foraging groups were once present all across Southern Africa, with a much larger distribution than the previously observed (Mitchell 2010; Barbieri, Vicente, et al. 2013). Their former presence over a wide geographic area has raised the possibility of the assimilation of these non-Bantu groups into farming communities through complex integration dynamics (Mitchell 2002), with the legacy of these events being recovered in contemporary Bantu-speaking populations (Sikora et al. 2011; Barbieri, Butthof, et al. 2013; Marks et al. 2014). It is within this context that the previously reported “South-Eastern” African component had been interpreted: The genetic signature of a pre-Bantu community assimilated into the Bantu speakers of Mozambique (Sikora et al. 2011). Archaeological evidence supports the idea that the dispersal of Bantu speakers was relatively fast along the Eastern part of Africa, which suggested reduced interactions between the Bantu-speaking populations and the ones previously present in the occupied areas (Alexander 1984). The change in the pace of migration occurred much further south, around Lesotho and South Africa, where the ecological conditions encountered were inappropriate for the crops used by these farming communities. The slowing of the process favoured more extended interaction between communities and possibly facilitated gene-flow (Bohannan and Plog 1967; Alexander 1984). The different admixture dynamics experienced by Bantu-speaking communities in different areas of South-Eastern Africa resulted in different degree of assimilation of hunter-gatherers and pastoralist communities (Marks et al. 2014).

The previously reported differentiation of the Mozambique sample from other Bantu-speaking populations was originally interpreted as evidence for a pre-Bantu genetic component in South-Eastern Africa (Sikora et al. 2011). When we reanalyzed the Mozambican data within a data set comprising several Southern African Bantu-speaking populations, the reported uniqueness of this population disappeared. In the second component of the PCA, Mozambique appears as part of a cluster of several sub-Saharan African populations speaking NC languages and distributed along a Northeast–Southwest continuum (fig. 3*b*). The ADMIXTURE components present in the Mozambican samples are the same as those of other East and South-Eastern African populations from Kenya, South Africa, and Lesotho (fig. 4*a*). The major component in Mozambique is also the major component in other populations from the South-Eastern region (green in fig. 4*a*), and is present at lower frequencies in other NC speakers. Importantly, the closer affinity of this component to the other NC component (NC-West) suggests a more recent common origin for the two NC clusters than any of the others. The pairwise *F*_ST_ population tree confirms the clustering of all NC-speaking populations, with the South-Eastern Bantu-speakers placed within the variation of other Bantu-speaking populations (red and green branches in fig. 5). Overall, is there any support for the presence of a predominantly non-Bantu component in South-Eastern Africa? Our results suggest some degree of stratification among Bantu-speaking populations which matches the West versus East NC linguistic split (Currie et al. 2013). The differential distribution of the two ADMIXTURE components in the NC populations, coupled with the gradual increase of the green component when moving from populations living in the west, to those living in the East and the Southeast, hints to a degree of differentiation among Bantu-speaking populations shaped by founder events and drift associated with their geographic dispersal rather than massive gene introgression from other groups (Ramachandran et al. 2005). No other components, known (Khoisan or RHG) or unknown, are detected within the Mozambican sample at significant level. Despite the lack of a major non-Bantu component in Mozambique, we note that other groups in Southern Africa are characterized by such kind of contribution, in this case Khoisan (fig. 4*a*) in agreement with previous reports of Khoisan admixture in Southern African Bantu-speaking populations (Schlebusch et al. 2012; Petersen et al. 2013). This is confirmed by *f3* analyses that highlight several admixture events involving African populations. However, we failed to identify any signature of admixture between Khoisan populations (or other hunter-gatherer groups) and Bantu speakers in Mozambique. In fact, none of the tests in the form *f3*(*Target; Pop A, Pop B*) gave significant or negative values. Furthermore, our CV analysis based on resampled data shows that the probability of having a false negative result is very low. Even though the absence of gene flow into the Mozambican sample here analyzed cannot be completely excluded given the low number of analyzed markers, we show that a high contribution from non-Bantu populations is highly unlikely in this population. We also noted that this might not be the case for all populations from this area as the analyzed sample is possibly not representative of the region as a whole. Sporadic highly divergent mtDNA and Y chromosome lineages have been reported in Zambia and Mozambique despite the lack of robust population signatures of admixture (Batini et al. 2011; Barbieri, Butthof, et al. 2013; Barbieri, Vicente, et al. 2013; Marks et al. 2014). Similarly, we cannot refute a scenario where admixture occurred in the past but left no signature in modern day populations. Future analysis of ancient DNA will be crucial to further refine the model discussed in this work. Unfortunately, the climatic conditions in Sub-Saharan Africa make the extraction of endogenous DNA still challenging but new methods developed in the last few years are promising as recently showed (Morris et al. 2014; Sirak et al. 2015). The observation of some degree of differentiation between Western and Eastern–South–Eastern Bantu speakers also suggests that higher SNP densities might provide the power to identify and distinguish populations belonging to the two Bantu linguistic branches, which could be of help in elucidating the demic pattern of Bantu speakers dispersal as well as reconstruct the African ancestry of recently admixed populations (de Filippo et al. 2012; Montinaro et al. 2015).

In conclusion, our results underline the role played by both the dispersal of Bantu-speaking groups and the interaction with previous inhabitants in shaping the genetic and cultural variation of Bantu-speaking populations of Southern Africa. Such combined effect has been suggested for other regions of Africa where Bantu-speaking groups are present (Patin et al. 2014). The integration of archaeological and linguistic data with a more complex demographic model is necessary to better understand the process through which languages, people, and technology were spread following the so-called “Bantu expansion” (Marks et al. 2014).

## Materials and Methods

### Samples

Saliva samples from unrelated individuals were collected in Namibia and Lesotho using the Oragene DNA collection kits (DNA Genotek, Inc., Ottawa, ON, Canada) and DNA was extracted according to manufacturer’s protocols. The samples presented here were collected during three field trips. The Basotho speakers were collected in Lesotho in 2009 (Marks et al. 2012, 2014); the Owambo, Mbukushu, and Kwangali were collected in Namibia in 2010 and 2012. All participants were healthy adults from whom appropriate informed consent was obtained. These investigations received ethical approval by the Oxford Tropical Research Ethics Committee, the Lesotho Ministry of Health and Social Welfare, the Lesotho Ministry of Local Government, the Lesotho Ministry of Tourism, Environment and Culture, and the Namibian Ministry of Health and Social Services. Ethnic and linguistic information about the donors, as well as their parents and grandparents if known, was also collected.

A total of 33 individuals (8 Basotho, 10 Owambo, 8 Mbukushu, and 7 Kwangali) were genotyped using the Illumina Human 610-Quad BeadChip (Illumina, San Diego, CA) (Basotho and Owambo) and the Human Omni5-Quad BeadChip (Illumina) (Mbukushu and Kwangali). The analyzed data described in this article are available on CC’s group website (https://capelligroup.wordpress.com/data/).

### Data Set

We combined our data with available genome-wide SNP genotypes from different African populations, using the software PLINK version 1.9 (Chang et al. 2015; Purcell and Chang 2015) (fig. 2).

The assembled data set was pruned through quality control (QC) filtering both SNPs and individuals. Each single population data set was initially filtered to remove SNPs and individuals with a call rate below 0.9. After the merge, an additional QC step was performed, removing SNPs and individuals with a call rate below 0.98. Related individuals up to the second degree were removed from our data set using the software KING (Manichaikul et al. 2010). To overcome the effects of markers in strong linkage disequilibrium, we removed SNPs with an *r*^2 ^> 0.4 using a sliding window of 200 SNPs, shifted at 25 SNP intervals (Behar et al. 2010).

To detect and remove samples with strong European admixture from our data set, the CEU data from HapMap Phase 3 (http://hapmap.ncbi.nlm.nih.gov/, last accessed July 30, 2015) (International HapMap Consortium 2003) were initially included in the analysis. We then performed an ADMIXTURE run (Alexander et al. 2009) with the default options and individuals characterized with more than 10% of the European component (for *K* = 4) were removed. The final data set was composed of a total of 868 individuals from 35 populations (International HapMap Consortium 2003, 2007; Li et al. 2008; Henn et al. 2011; Sikora et al. 2011; Schlebusch et al. 2012; May et al. 2013; Petersen et al. 2013) typed on 1,747 SNPs (fig. 2 and supplementary table S1, Supplementary Material online). Similar number of SNPs has been successfully used to recover evident and robust patterns of genetic diversity in African populations (Sikora et al. 2011).

### Statistical Analysis

Population genetic structure was initially explored through PCA with PLINK software version 1.9 (Chang et al. 2015; Purcell and Chang), and we used ADMIXTURE (version 1.23) (Alexander et al. 2009) to further explore genetic variation, using a range of putative source clusters (*K*) from 2 to 20. The most supported value of *K* was estimated using the CV procedure, as implemented in ADMIXTURE (Alexander et al. 2009; Alexander and Lange 2011). We computed *F*_ST_ between the components to assess genetic differentiation (Alexander et al. 2009). The spatial distribution of the ADMIXTURE components (*K* = 6, see Results) was visualized by plotting the fraction of each component in the analyzed populations on a map with the interpolation plugin of the QGIS software, using the Inverse Distance Weighting method with a distance coefficient of 3 (QGIS Development Team 2014). Weir and Cockerham’s pairwise *F*_ST_ between populations was calculated using the R package StAMPP (Weir and Cockerham 1984; Pembleton et al. 2013). The R packages stats and ape were used to build and visualize, respectively, a hierarchical tree with these values, using the complete linkage method (Paradis et al. 2004; Legendre P and Legendre L 2012; R Core Team 2014).

Given the fact that ADMIXTURE analysis is not a formal text of gene-flow, we carried out a *f3* test (Reich et al. 2009) for windows of 100 markers using the threepop companion software in the TreeMix suite (Pickrell and Pritchard 2012), and reported *f3* statistics characterized by a value lower than −3. The *f3* statistics has been demonstrated to be robust, even with ascertainment bias (Patterson et al. 2012). Briefly, in a *f3* test with the form *f3*(*Target; Pop A, Pop B*) a significantly negative value of the statistic highlights a complex phylogeny of the Target population, that has a certain amount of ancestry from populations related to A and B. However, a positive *f3* value does not necessarily imply the absence of admixture. For this reason, and given the low number of markers used in this analysis, we performed a *f3*(*Target; Yoruba, Pop 1*) test on 100 simulated samples composed by Yoruba and Pop1, with proportion alpha and 1-alpha respectively, where Pop1 is represented by Juhoansi, Sandawe or Mbuti, and alpha **∈**{0.05, 0.10, . . . , 0.95}.

## Supplementary Material

Supplementary figures S1–S6 and tables S1–S3 are available at *Genome Biology and Evolution* online (http://www.gbe.oxfordjournals.org/).

Supplementary Data
