# Supplementary material for: Genome-Wide SNP Analysis of Southern African Populations Provides New Insights into the Dispersal of Bantu-Speaking Groups
Source: Genome Biol Evol. 2015 Sep 11;7(9):2560–8. doi: 10.1093/gbe/evv164 (PMC4607521; doi:10.1093/gbe/evv164)
Supplement: Supplementary Data [file supp_7_9_2560__index.html]

Genome-wide SNP analysis of Southern African populations provides new insights into the dispersal of Bantu speaking groups — Genome-Wide SNP Analysis of Southern African Populations Provides New Insights into the Dispersal of Bantu-Speaking Groups — Supplementary Data 

# Genome-Wide SNP Analysis of Southern African Populations Provides New Insights into the Dispersal of Bantu-Speaking Groups

## Supplementary Data

files

- Supplementary Data - zip file
