# Supplementary material for: Genome-Wide SNP Analysis of Southern African Populations Provides New Insights into the Dispersal of Bantu-Speaking Groups
Source: Genome Biol Evol. 2015 Sep 11;7(9):2560–8. doi: 10.1093/gbe/evv164 (PMC4607521; doi:10.1093/gbe/evv164)
Supplement: Supplementary Data [file supp_evv164_suppl_data.zip › Supplementary Material - Table S1 Figures S1-S6.pdf]

Electronic Supplementary Material

Table S1 – Populations from other studies used in this work, including number of individuals initially available and used after QC.

Population	Reference	n	
		Initial	Included
Khwe	Schlebusch *et al*. 2012	17	17
Nama	Schlebusch *et al*. 2012	20	12
Xun_A	Petersen *et al*. 2013	14	13
Xun_B	Schlebusch *et al*. 2012	19	18
GuiGana	Schlebusch *et al*. 2012	15	14
Juhoansi_A	Petersen *et al*. 2013	19	19
Juhoansi_B	Schlebusch *et al*. 2012	18	18
Juhoansi_C	Li *et al*. 2008	5	5
Karretjie	Schlebusch *et al*. 2012	20	11
Khomani	Schlebusch *et al*. 2012	39	16
MbutiPygmy	Li *et al*. 2008	13	13
BiakaPygmy	Li *et al*. 2008	21	21
Mandenka	Li *et al*. 2008	22	22
Yoruba_A	Frazer *et al*. 2007	203	101
Yoruba_B	Li *et al*. 2008	21	21
SWBantu	Schlebusch *et al*. 2012	12	9
Luhya	Frazer *et al*. 2007	110	81
KenyaBantu	Li *et al*. 2008	11	8
Anuak	Pagani *et al*. 2012	23	21
Gumuz	Pagani *et al*. 2012	19	13
SouthSudan	Pagani *et al*. 2012	24	21
AriBlacksmith	Pagani *et al*. 2012	17	15
AriCultivator	Pagani *et al*. 2012	24	22
Maasai	HapMap	184	96
Sandawe	Henn *et al*. 2011	28	23
Hadza	Henn *et al*. 2011	17	3
MozambiqueBantu	Sikora *et al*. 2011	180	69
SouthAfricaBantu	Li *et al*. 2008	8	8
SEBantu	Schlebusch *et al*. 2012	20	19
SowetoBantu	May *et al*. 2013	94	91
amaXhosa	Petersen *et al*. 2013	15	15
		1252	835

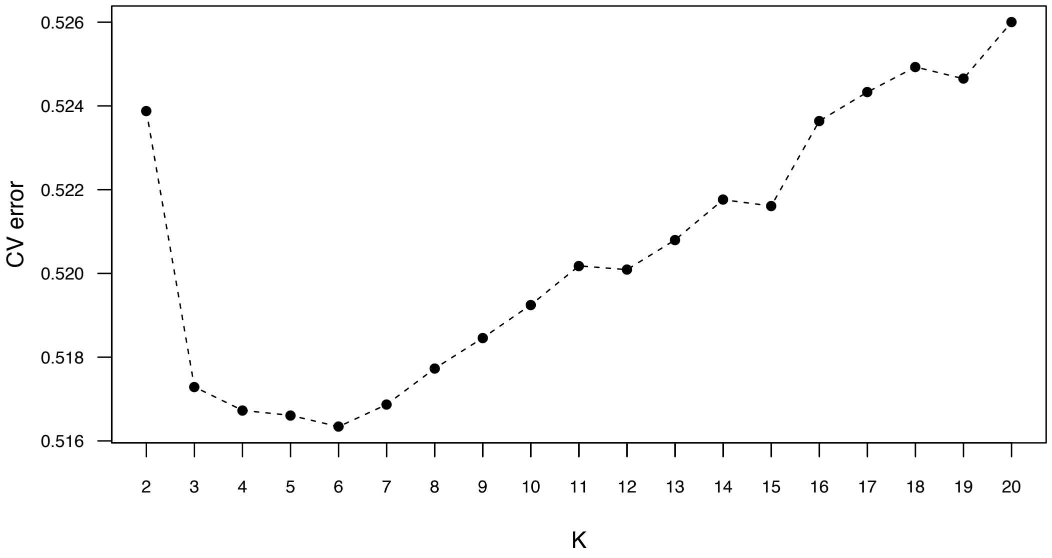

Fig. S1 – CV plot for the ADMIXTURE analysis. Lowest value was observed for *K*=6 (0.51634).

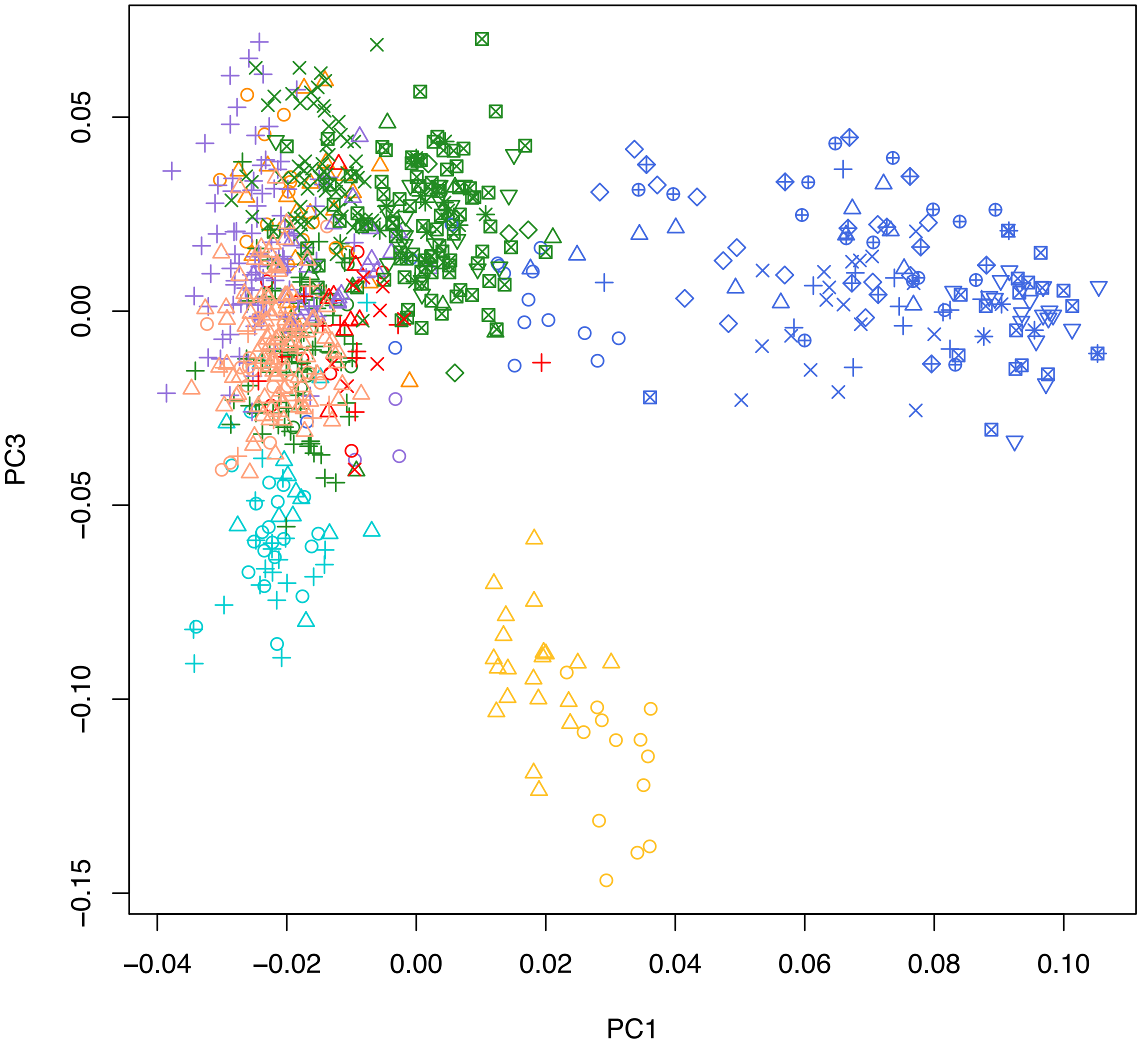

Fig. S2 – Principal Components (PC) plot for the first and third components. Colours and symbols as in Figure 3.

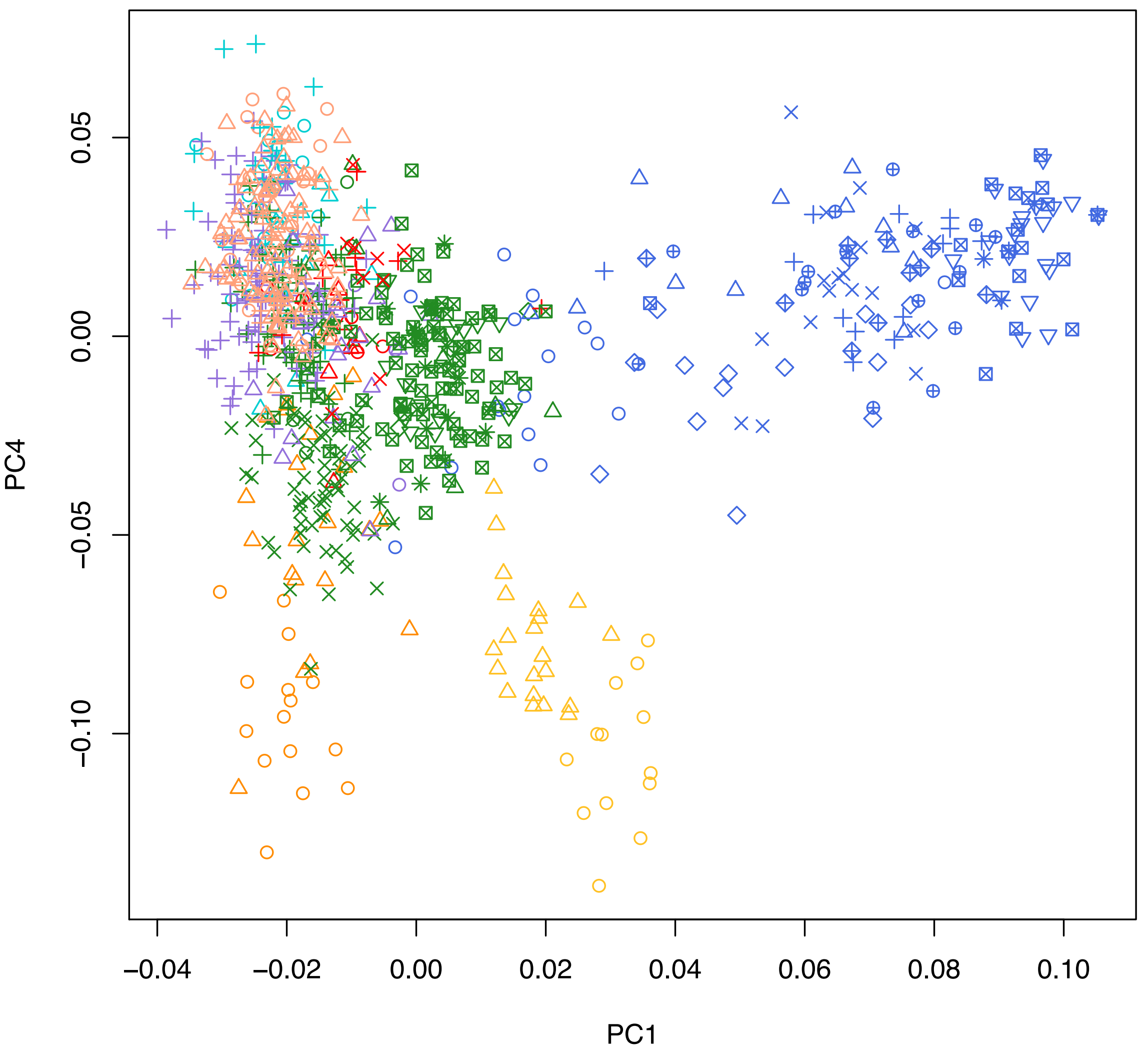

Fig. S3 – Principal Components (PC) plot for the first and fourth components. Colours and symbols as in Figure 3.

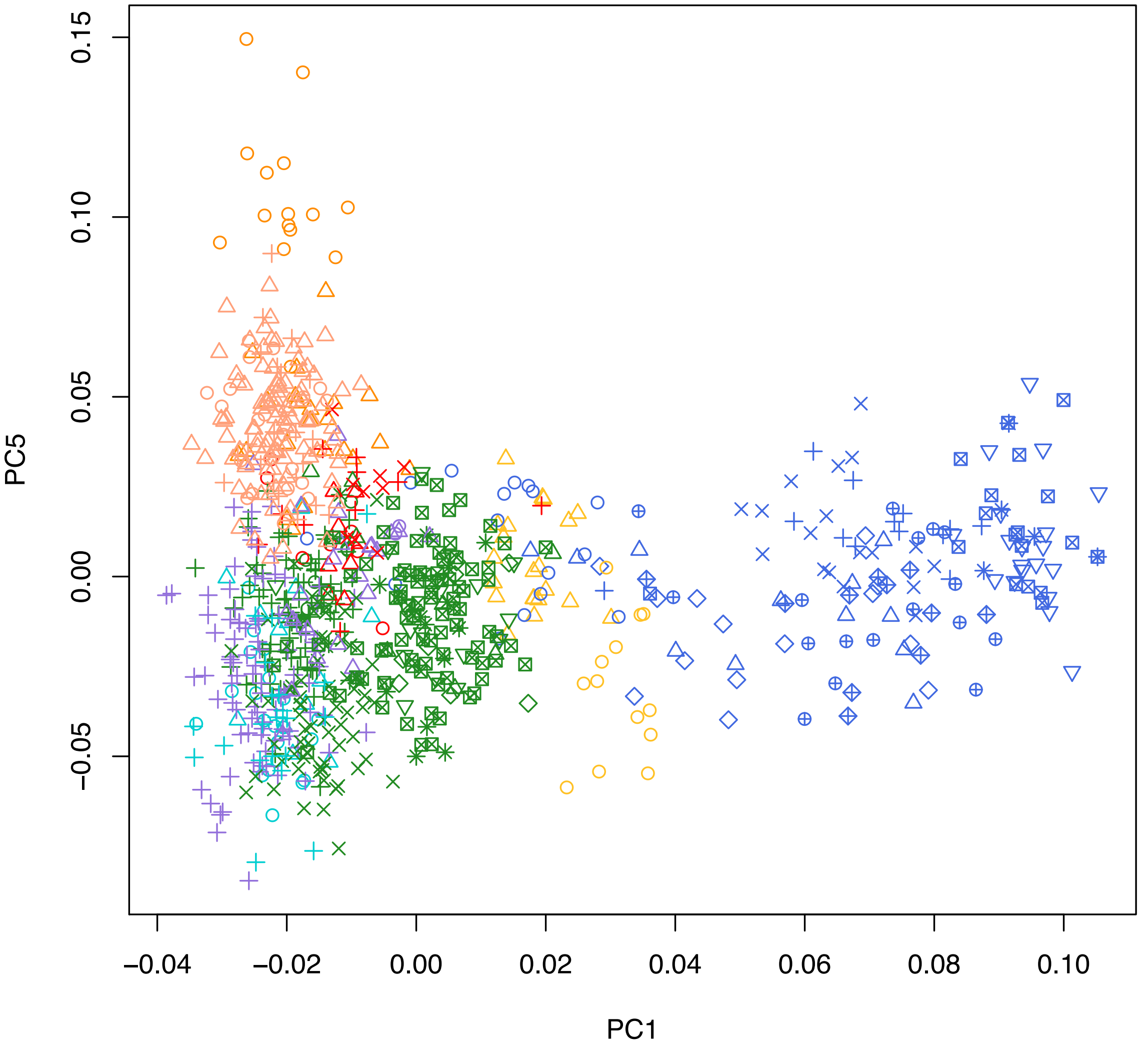

Fig. S4 – Principal Components (PC) plot for the first and fifth components. Colours and symbols as in Figure 3.

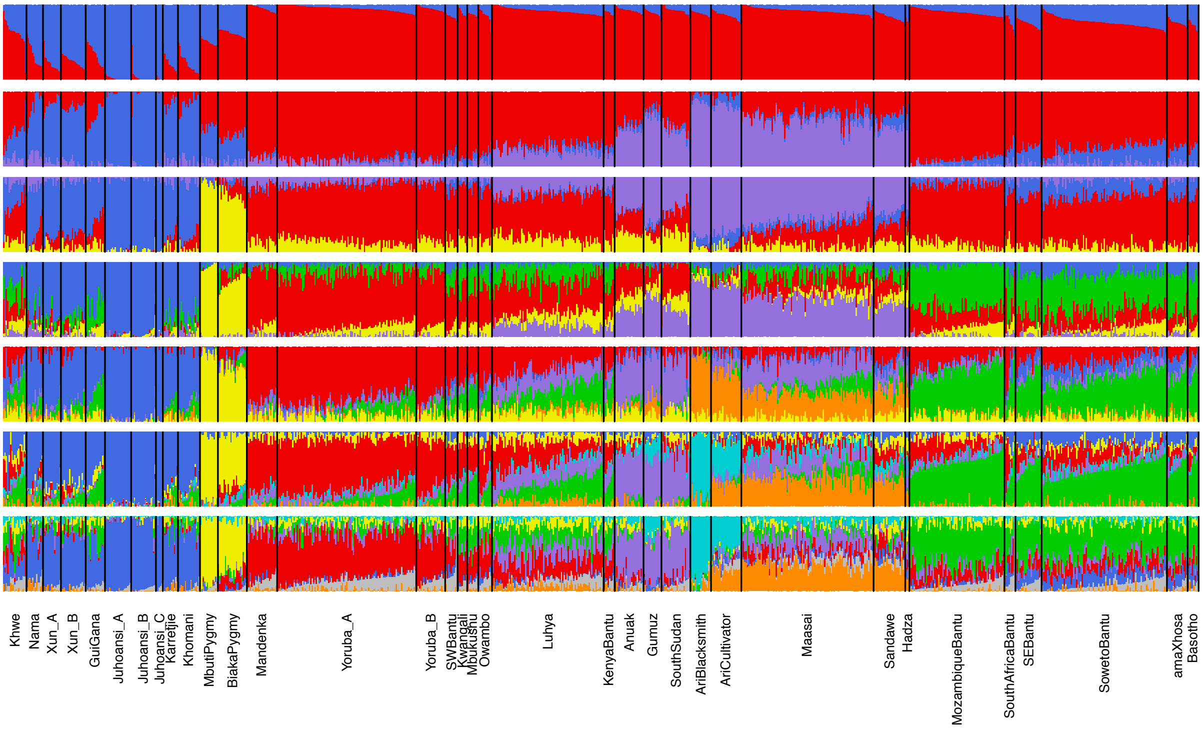

Fig. S5 – ADMIXTURE plots from K=2 to K=8.

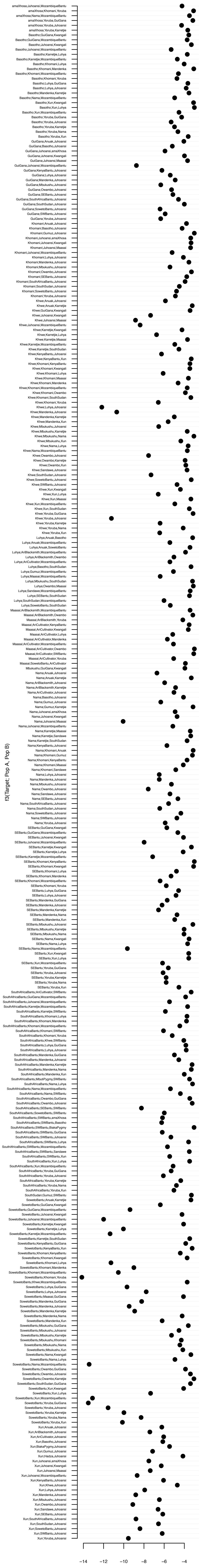

Fig. S6 – Significant comparisons (Z-score < -3) for the f3 tests between all possible trios of populations in our dataset.
